# On the escape from potentials with two exit channels

**DOI:** 10.1038/s41598-019-49765-y

**Published:** 2019-09-11

**Authors:** Juan F. Navarro

**Affiliations:** University of Alicante, Department of Applied Mathematics, Alicante, 03690 Spain

**Keywords:** Applied mathematics, Computational science

## Abstract

The aim of this paper is to investigate the escape dynamics in a Hamiltonian system describing the motion of stars in a galaxy with two exit channels through the analysis of the successive intersections of the stable and unstable manifolds to the main unstable periodic orbits with an adequate surface of section. We describe in detail the origin of the spirals shapes of the windows through which stars escape.

## Introduction

When we look up at the night sky, the stars appear fixed but, in fact, stars move through our galaxy although the changes in their positions on the sky are too small and slow to be appreciated over human timescales. These changes in the stars positions were first discovered in the eighteenth century by Edmond Halley, through the comparison of stellar catalogues from his time to a catalogue compiled by Hipparchus some two thousand years before. Nowadays, the motion of a star can be detected with a few years’ worth of high-precision astrometric observations. Some questions involving the nature of the motion of a star in a galaxy may arise: What kind of motions are possible in a galactic potential? How can we determine if a star can escape or not from the galaxy? The last question has focused the interest of many researchers during the last decades^1–17^. The most of these efforts have been devoted to the numerical exploration of galactic systems by integrating sets of initial conditions regularly distributed in dense grids defined in an adequate surface of section. For each initial condition, the equations of motion must be integrated along a maximum time of integration or until the star leaves the potential. The maximum integration time is taken large enough to be sure that all orbits have enough time to escape. Even though this procedure gives information about the location of the basins of escape through the different exits in the surface of section considered, they can not describe the structure of the limiting curves of the regions of escape. In 1990, Contopoulos^9^ explored the phenomenon of escape in two conservative Hamiltonian systems representing the central part of a perturbed galaxy, with quadruple symmetry and four openings to infinity, and one axis symmetry and two openings to infinity, respectively. In both cases, there is an unstable periodic orbit (called “Lyapunov” orbit) located at every opening of the potential well. Contopoulos found that the asymptotic curves of the various unstable periodic orbits intersect at a complicated set of homoclinic and heteroclinic points, and govern the escape to infinity from the potential well. Navarro and Henrard^12^ analyzed the structure of the windows of escape for the galactic system presenting four openings in the potential well, describing deeply the complex layered nature of the spirals obtained in Poincaré sections. There are “first-order” infinite spirals as those described by Contopoulos, but also “second order” spirals which are composed of an infinity of layers, “third order” spirals formed by an infinity of second order spirals, and so on. In 2017, Navarro^13^ investigated the shape of the windows of escape to infinity from a galaxy modeled by a bi-symmetrical harmonic oscillator perturbed with quartic terms, finding the same mechanism governing the escape from the potential well.

Even though the infinite layered structure of the spirals is well-known, it has never been unveiled with enough detail. The aim of this paper is to analyze the composition of this complex structure of infinite spirals to shed light on the geometry of the windows through which stars may escape from a galactic potential. This way leads to a better understanding of the properties of the escape of a particle from a galactic system. When the energy *h* of a star in a galaxy is larger than a certain critical value *h*_*c*_, the star may escape to infinity. But not all stars with energy larger than the critical value escape from the galactic potential. There are many stars which remain trapped around a stable periodic orbit. In order to determine the escaping sets we must calculate the asymptotic manifolds to the Lyapunov orbits in the phase space. The intersection of the stable manifold with a surface of section originates a closed curve called a “limiting asymptotic curve”. Any orbit with initial condition inside this limiting curve escapes to infinity without intersecting again the surface of section. If we consider the successive intersections of the asymptotic manifold with the surface of section, we obtain a complicated structure, that we will describe in detail throughout this paper.

One way to study the problem of escapes from a galaxy is by using a simple model. We will analyze the following galactic potential,

1 $$W(x,y)=-\frac{1}{2}({x}^{2}+{y}^{2})+\mu x{y}^{2},$$

where *μ* > 0. This potential presents two channels of escape. In 2014, Zotos^16^ performed a numerical exploration of this system, locating the basins of escape towards the different windows and unveiling their relation with the times of escape of the orbits. The Hamiltonian corresponding to potential (1) reads

2 $$H=\frac{1}{2}({\dot{x}}^{2}+{\dot{y}}^{2})-W(x,y),$$

that is

3 $$H=\frac{1}{2}({\dot{x}}^{2}+{\dot{y}}^{2})+\frac{1}{2}({x}^{2}+{y}^{2})-\mu x{y}^{2}.$$

The equations of motion are given by

4 $$\begin{array}{ccccc}\ddot{x} & = & {W}_{x}(x,y) & = & -x+\mu {y}^{2},\\ \ddot{y} & = & {W}_{y}(x,y) & = & -y+2\mu xy.\end{array}$$

First, we will compute the stable and unstable manifolds to the periodic orbits located at the two exit channels of the potential well, in order to show that these structures, composed of infinite spirals, governs the rate of escape. We will demonstrate that there are different levels of infinitely winding spirals, each of these level embedded into the next one. It is also necessary to understand how the geometry of these different levels of spirals are interrelated to have a deep knowledge about the shape and size of the regions of the phase space leading to escape. We will proceed by calculating the intersections of the asymptotic manifolds with a surface of section, as it has been done previously by Contopoulos and coworkers^9,10^ and Navarro and Henrard^12^. In our investigation, we will take as surface of section the plane *y* = 0.

## Curves of Zero Velocity

**Figure 1:**
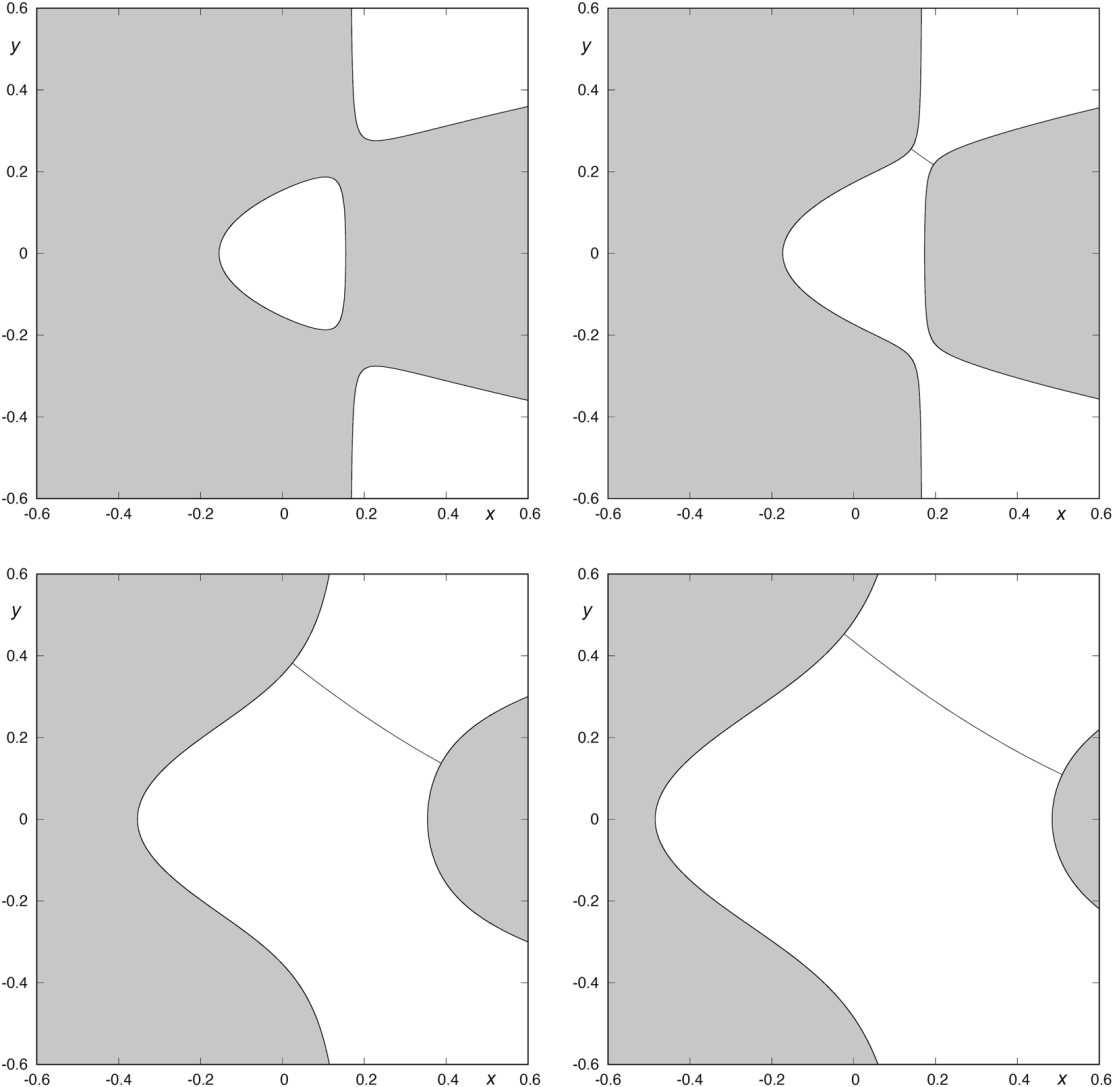
Curves of zero velocity for *μ* = 3, and different values of the energy: *h*_1_ = 0.012, *h*_2_ = 0.01504, *h*_3_ = 0.062802 and *h*_4_ = 0.11760. As the energy of the system grows, the windows of the potential well become wider. The guardian orbit is the almost straight line barring the opening.

The energy of the system is a constant of motion, so it is possible to obtain an equation relating the velocity of the particle to its position. If we consider a fixed value of the energy, we can construct the curves in the plane on which velocity vanishes. These are the so-called curves of zero velocity. Figure 1 shows some contours of the curves of zero velocity for several values of the energy. These curves describe the boundary between regions of possible motion and forbidden regions. Depending on the value of the energy, the system exhibit different types of behavior. There is a critical value of the energy (*h*_*c*_) such that, for larger values of *h*, the curves of zero velocity are open and test particles may escape. For *h* < *h*_*c*_, the curves are effectively closed and particles originated from the central potential region are confined there. If the energy of a star is larger than *h*_*c*_, the curves of zero velocity present two openings (Fig. 1). At the openings we find two unstable periodic orbits, the so-called Lyapunov orbits. Due to the symmetries of the potential, the well opens up, for values of the energy larger than the critical value *h*_*c*_, at two places along the lines defined by $y=\pm \sqrt{2}x$.

We have computed the critical value by using the method described by Caranicolas^18^, obtaining

$${h}_{c}=\frac{1}{8{\mu }^{2}}.$$

The curves of zero velocity of the system (4) are given by the relation

5 $$f(x,y)=h-\frac{1}{2}({x}^{2}+{y}^{2})+\mu x{y}^{2}=0.$$

The points of the *xy* plane where the curve of zero velocity opens are the saddle-points of (5). To compute them, we must first solve the system

6 $$\frac{\partial f}{\partial x}=\mu {y}^{2}-x=0,\,\frac{\partial f}{\partial y}=2\mu xy-y=0.$$

The solutions of (6) are the critical points of (5). The saddle-points of (5) are the solutions of (6) satisfying the condition

$$S=(\frac{{\partial }^{2}f}{\partial {x}^{2}})(\frac{{\partial }^{2}f}{\partial {y}^{2}})-{(\frac{{\partial }^{2}f}{\partial x\partial y})}^{2} < 0.$$

In addition to the trivial solution *x* = *y* = 0, there are two saddle-points in the *xy* plane, given by

7 $$x=\frac{1}{2\mu },\,{y}^{2}=\frac{1}{2{\mu }^{2}}.$$

The substitution of the solution (7) into Eq. (5), leads to the critical value

$${h}_{c}=\frac{1}{8{\mu }^{2}}.$$

In our analysis, we will take *μ* = 3. The critical value of the energy associated to this value of *μ* is ${h}_{c}=1/72\simeq 0.0138888888$. For each larger value of *h*, there is a Lyapunov orbit bridging the opening, bouncing back and forth between the two “walls” of the pass (see Fig. 1). The most important property of Lyapunov orbits is that any orbit crossing them outwards moves always outwards and escape from the system. These unstable Lyapunov orbits can be considered as the guardians of the pass between the inner region and the outside region, as the asymptotic orbits to them compose the limits of the sets of escaping orbits.

The aim of this investigation is to clarify the properties of the escape for values of the energy close to the critical value *h*_*c*_. To this end, we will analyze the geometry of the asymptotic curves to the Lyapunov orbits for a value of the energy close enough to *h*_*c*_.

## Computation of the Stable and Unstable Manifolds

**Figure 2:**
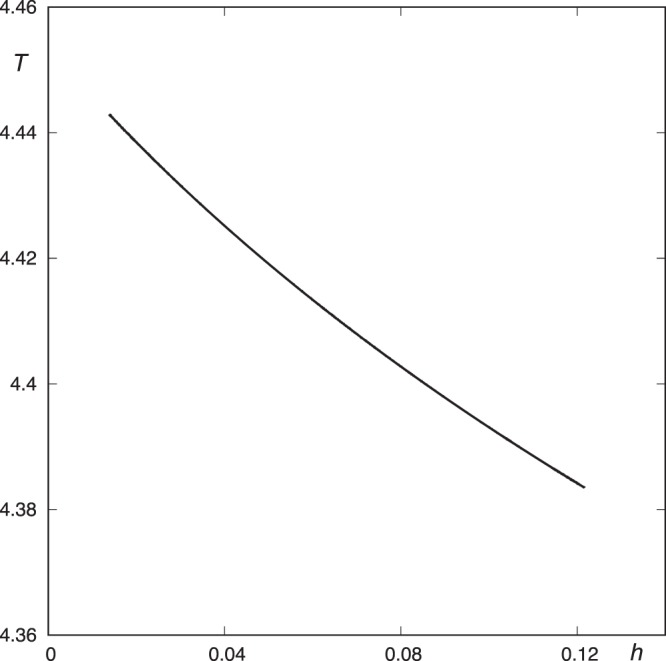
Continuation of the family of unstable periodic orbits guarding the potential well.

As we are interested in the determination of the escaping sets, we must compute the asymptotic manifolds to the Lyapunov orbits. We have obtained the initial conditions of the upper Lyapunov orbit for a value of the energy slightly larger than the critical value through a geometric approach (Table 1). Then, we have applied the procedure by Deprit and Henrard^19^ to compute the family of periodic orbits taking the energy as parameter. The result of this continuation is shown in Fig. 2.

**Table 1:** Initial conditions of the periodic orbit.

*x*_0_	${\dot{x}}_{0}$	*T*	*h*
0.16717634574128063	0.03919913312750965	4.44203388800269796	0.01504031098093976

As mentioned in the introduction, the stable asymptotic curves to the “guardian” Lyapunov orbits form the boundaries of the escape windows we are interested in. In order to compute the initial part of these asymptotic curves, we follow the scheme proposed by Deprit and Henrard^20^. Let (x^*^(*t*), y^*^(*t*)) be a periodic orbit of equations (4), and *T* be its minimal period. As the system is conservative and Hamiltonian, two of its multipliers are equal to +1; the other two are of the form (*ρ*,1/*ρ*), where *ρ* is a nonzero complex number. If *ρ* is real and |*ρ*| ≠ 1, then Poincaré^21^ established that the periodic orbit is unstable, and there exists a real number *a* > 0 such that *ρ* = *e*^*aT*^. We will refer to *a* as the characteristic exponent of the periodic orbit (x^*^(*t*), y^*^(*t*)).

Poincaré showed that there are two one-parameter families of orbits associated to the unstable periodic orbit: a one-parameter family of orbits which tend asymptotically to the periodic orbit as the time *t* goes to +∞ (incoming asymptotic orbits), and a one-parameter family of orbits which tend asymptotically to the periodic orbit as *t* goes to −∞ (outgoing asymptotic orbits).

Thus, the incoming asymptotic orbits to a periodic orbit (x^*^(*t*), y^*^(*t*)) of period *T* are represented by the series

8 $$x(t,\varepsilon )={x}^{\ast }(t)+\varepsilon u(t),\,y(t,\varepsilon )={y}^{\ast }(t)+\varepsilon v(t),$$

where

9 $$\begin{array}{ccc}u(t) & = & \sum _{j\ge 1}\,{\varepsilon }^{j-1}{e}^{-jat}{x}_{j}(t),\\ v(t) & = & \sum _{j\ge 1}\,{\varepsilon }^{j-1}{e}^{-jat}{y}_{j}(t),\end{array}$$

for any integer *j* ≥ 1, the coefficients *x*_*j*_(*t*) and *y*_*j*_(*t*) are periodic with period *T*. The outgoing asymptotic orbits are represented by similar series, replacing *a* by −*a*. When *ε* is equal to zero, we recover the periodic orbit. Moreover, the asymptotic solutions (8) lie on the same energy manifold as the periodic orbit and, consequently, the series (9) represent an isoenergetic displacement of the periodic orbit.

Ths substitution of the series (8) into the differential equations (4) leads to

10 $$\begin{array}{ccc}\ddot{u} & = & {W}_{xx}^{\ast }u+{W}_{xy}^{\ast }v+\sum _{i\ge 1}\,{\varepsilon }^{i-1}\sum _{j\ge 0}\,{c}_{i}^{j}{(\frac{{\partial }^{i}W}{\partial {x}^{j}\partial {y}^{i-j}})}^{\ast }{u}^{j}{v}^{i},\\ \ddot{v} & = & {W}_{yx}^{\ast }u+{W}_{yy}^{\ast }v+\sum _{i\ge 1}\,{\varepsilon }^{i-1}\sum _{j\ge 0}\,{c}_{i}^{j}{(\frac{{\partial }^{i+j+1}W}{\partial {x}^{j}\partial {y}^{i-j}})}^{\ast }{u}^{j}{v}^{i}\mathrm{.}\end{array}$$

Here, f^*^ denotes the function *f* evaluated along the periodic solution, that is, f^*^(*t*) = *f*(x^*^(*t*), y^*^(*t*)). Expanding *u*(*t*) and *v*(*t*) in powers of *ε*, the equation (10) lead to a recursive set of linear equations for the coefficients *x*_*k*_(*t*) and *y*_*k*_(*t*),

11 $$\begin{array}{ccc}{k}^{2}{a}^{2}{x}_{k}+2ka{\dot{x}}_{k}+{\ddot{x}}_{k} & = & {W}_{xx}^{\ast }\,{x}_{k}+{W}_{xy}^{\ast }\,{y}_{k}+{\Phi }_{k}(t),\\ {k}^{2}{a}^{2}{y}_{k}+2ka{\dot{y}}_{k}+{\ddot{y}}_{k} & = & {W}_{xy}^{\ast }\,{x}_{k}+{W}_{yy}^{\ast }\,{y}_{k}+{\Psi }_{k}(t),\end{array}$$

where Φ_*k*_(*t*) and Ψ_*k*_(*t*) are periodic functions corresponding to the contributions of the summations over *j* in (11) multiplied by *e*^*jat*^. These contributions can readily be computed at order *k* and are periodic if the ${x}_{\ell }(t)$ and ${y}_{\ell }(t)$ (for $\ell  < k$) are known and periodic. As a matter of fact, we do not need to know Φ_*k*_(*t*) and Ψ_*k*_(*t*) explicitly as functions of the time. As these coefficients have to be interpolated in Fourier series, it is enough to compute them at equally spaced instants along the period. Some details on the computation of the solution to (11) can be found in references ^12,13^.

## The Boundaries of the Escape Windows

We have integrated numerically and backward (using recurrent power series of order 20) some 1000000 orbits belonging to the stable manifold to the first quadrant Lyapunov orbit, until they cross the hyperplane *y* = 0 (with $\dot{y} > 0$). The initial conditions of this manifold have been computed by keeping the value of *εexp*(*at*) fixed at 0.0001 and choosing 1000000 equidistant values of the initial time *t*^22^.

In order to perform the analysis of the boundaries of the escape windows, we will introduce some nomenclature. Throughout the course of this paper, *ϕ*_*U*_(*t*) and *ϕ*_*L*_(*t*) denote the upper-right unstable periodic orbit, and the lower-right unstable periodic orbit, respectively. *W*_*s,ν*_(*ϕ*) denotes the *ν*-th intersection of the stable manifold to the unstable periodic orbit *ϕ* with the surface of section *y* = 0, and *Wu,ν*(*ϕ*) denotes the *ν*-th intersection of the unstable manifold to the unstable periodic orbit *ϕ* with the surface of section *y* = 0. Here, $\nu \in {\mathbb{N}}$.

In the following, we will show the connection between the asymptotic manifolds to *ϕ*_*U*_(*t*) and *ϕ*_*L*_(*t*) for a value of the energy slightly larger than the critical value, *h* = 0.015040.

**Figure 3:**
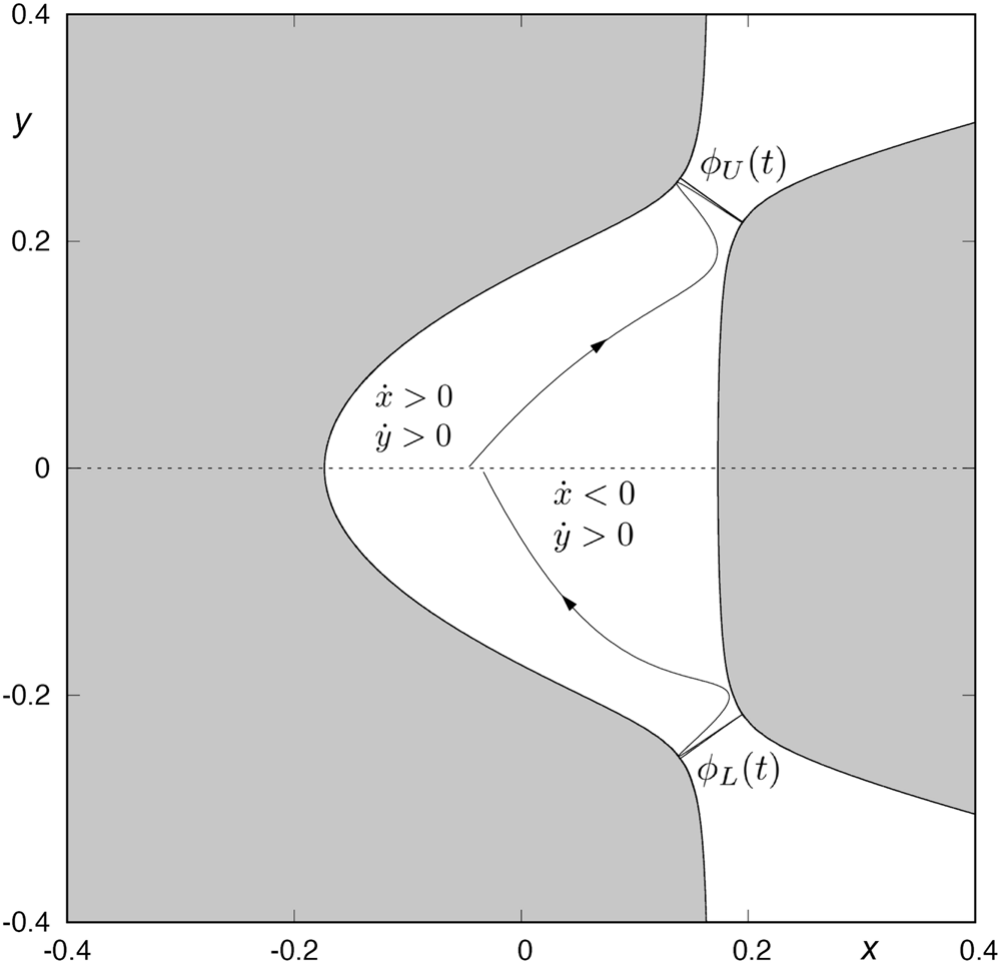
An orbit belonging to the stable manifold to the first quadrant periodic orbit *ϕ*_*U*_(*t*) integrated backwards until its intersection with the surface of section *y* = 0, and an orbit belonging to the unstable manifold to the first quadrant periodic orbit *ϕ*_*L*_(*t*) integrated backwards until its intersection with the surface of section *y* = 0. Here, *h* = 0.01504.

In Fig. 3, we show two orbits: the first one, belonging to the stable manifold to the first quadrant periodic orbit *ϕ*_*U*_(*t*), has been integrated backward until its intersection with the surface of section *y* = 0. The second one, belonging to the unstable manifold to the fourth quadrant periodic orbit *ϕ*_*L*_(*t*), has been integrated forward until its intersection with the same surface of section *y* = 0.

**Figure 4:**
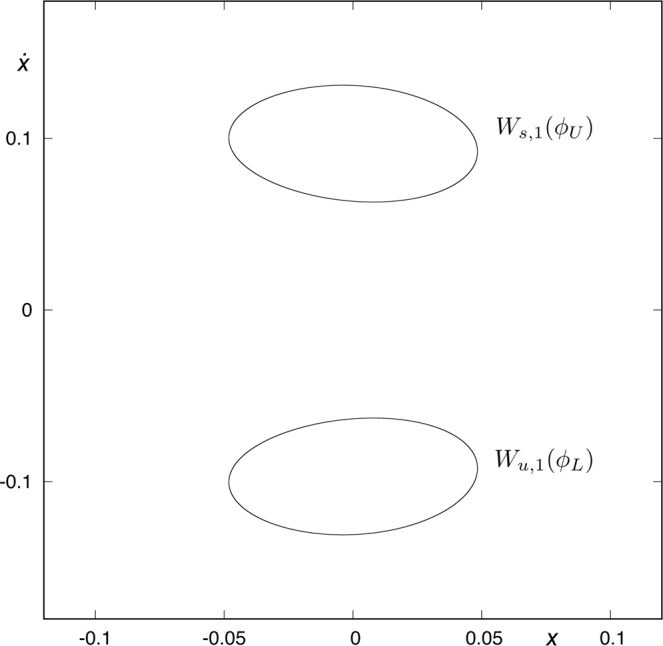
*W*_*s*,1_(*ϕ*_*U*_(*t*)) and *W*_*u*,1_(*ϕ*_*L*_(*t*)) for *h* = 0.01504. Both sets have an empty intersection, so we can not find any orbit coming from the lower-right infinity and escaping through the upper-right window after just one intersection with the surface of section.

Figure 4 shows the first intersection of the stable manifold to the periodic orbit *ϕ*_*U*_(*t*) with the surface of section *y* = 0, that is, *W*_*s*,1_(*ϕ*_*U*_), as well as the first intersection of the unstable manifold to the periodic orbit *ϕ*_*L*_(*t*) with the same surface of section, *W*_*u*,1_(*ϕ*_*L*_). We observe that these two sets, *W*_*s*,1_(*ϕ*_*U*_) and *W*_*u*,1_(*ϕ*_*L*_), do not intersect,

$${W}_{s,1}({\varphi }_{U})\cap {W}_{u,1}({\varphi }_{L})=\varnothing .$$

The points belonging to *W*_*s*,1_(*ϕ*_*U*_) intersect the surface of section *y* = 0 with $\dot{y} > 0$ and $\dot{x} > 0$, while the points in *W*_*u*,1_(*ϕ*_*L*_) intersect *y* = 0 with $\dot{y} > 0$ and $\dot{x} < 0$. Orbits starting inside *W*_*s*,1_(*ϕ*_*U*_) are going to the upper-right infinity, while orbits starting inside *W*_*u*,1_(*ϕ*_*L*_) are coming from the lower-right infinity. As *W*_*s*,1_(*ϕ*_*U*_) ∩ *W*_*u*,1_(*ϕ*_*L*_) = $\varnothing$, we can not find any orbit coming from the lower-right infinity and escaping through the upper-right window after just one intersection with the surface of section.

On the one hand, the second intersection of the stable manifold to the periodic orbit *ϕ*_*U*_(*t*) with the surface of section *y* = 0 takes place with a negative value of $\dot{y}$, so there is no intersection between *W*_*s*,2_(*ϕ*_*U*_) and *W*_*u*,1_(*ϕ*_*L*_). The same occurs between *W*_*s*,2*ν*_(*ϕ*_*U*_) and *W*_*u*,1_(*ϕ*_*L*_), for any $\nu \in {\mathbb{N}}$.

On the other hand, we have found that *W*_*s*,2*ν+*1_(*ϕ*_*U*_) ∩ *W*_*u*,1_(*ϕ*_*L*_) = $\varnothing$, for *ν* = 1, …, 6, and

$${W}_{s\mathrm{,15}}({\varphi }_{U})\cap {W}_{u\mathrm{,1}}({\varphi }_{L})\ne \varnothing \mathrm{.}$$

**Figure 5:**
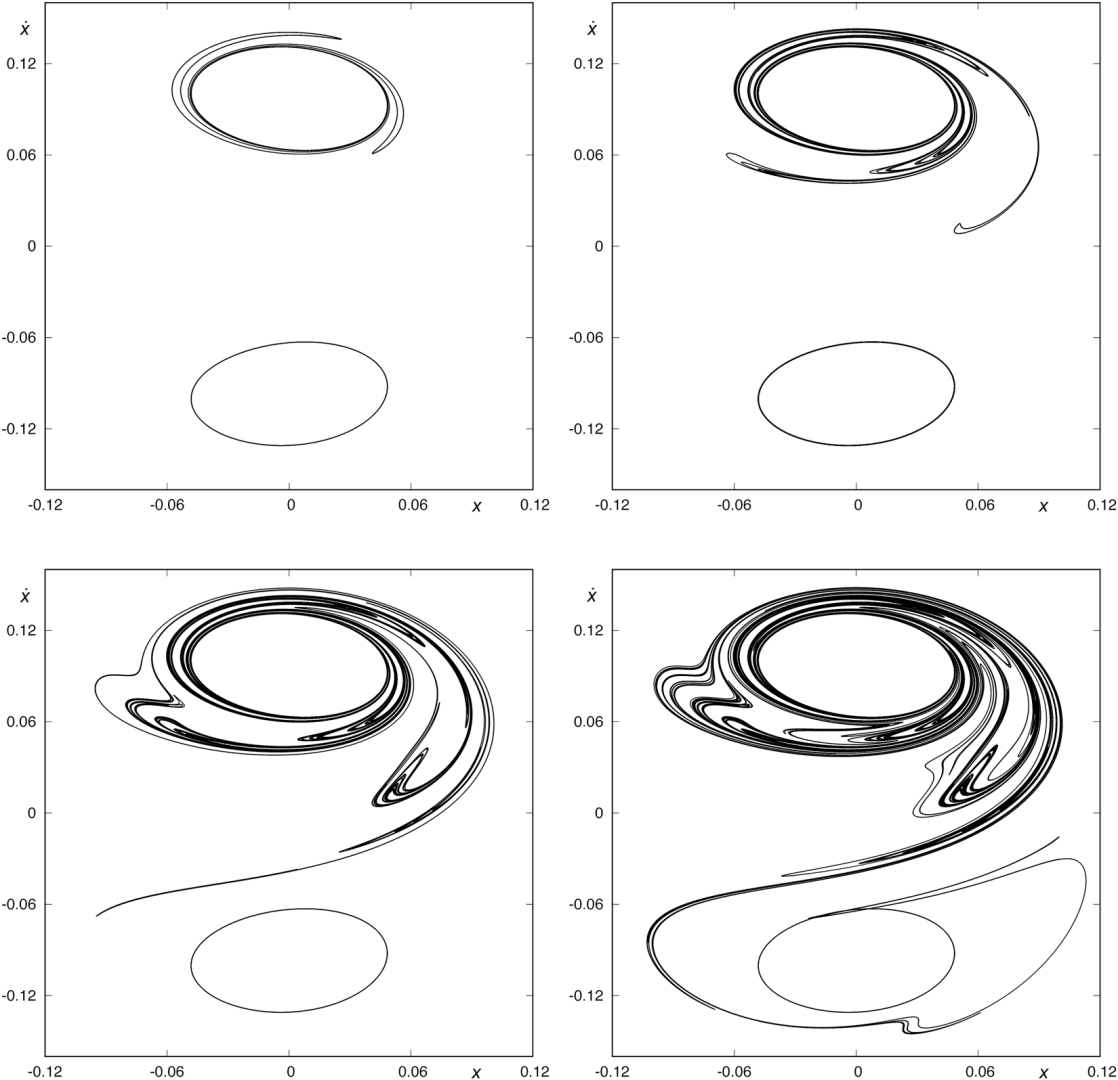
*W*_*s*,3_(*ϕ*_*U*_(*t*)) and *W*_*u*,1_(*ϕ*_*L*_(*t*)) (upper-left panel), *W*_*s*,7_(*ϕ*_*U*_(*t*)) and *W*_*u*,1_(*ϕ*_*L*_(*t*)) (upper-right panel), *W*_*s*,11_(*ϕ*_*U*_(*t*)) and *W*_*u*,1_(*ϕ*_*L*_(*t*)) (lower-right panel), and *W*_*s*,11_(*ϕ*_*U*_(*t*)) and *W*_*u*,1_(*ϕ*_*L*_(*t*)) (lower-right panel), for *h* = 0.01504. In the first three cases, the asymptotic curves to *ϕ*_*U*_ and *ϕ*_*L*_ have an empty intersection. However, *W*_*s*,11_(*ϕ*_*U*_(*t*)) ∩ *W*_*u*,1_(*ϕ*_*L*_(*t*)) ≠ $\varnothing$, so we can find orbits coming from the lower-right infinity and escaping through the upper-right window after fifteen intersections with the surface of section *y* = 0. We can also conclude that there are no orbits coming from the lower-right infinity and escaping through the upper-right window intersecting the axis *y* = 0 a number of times smaller than 15.

**Figure 6:**
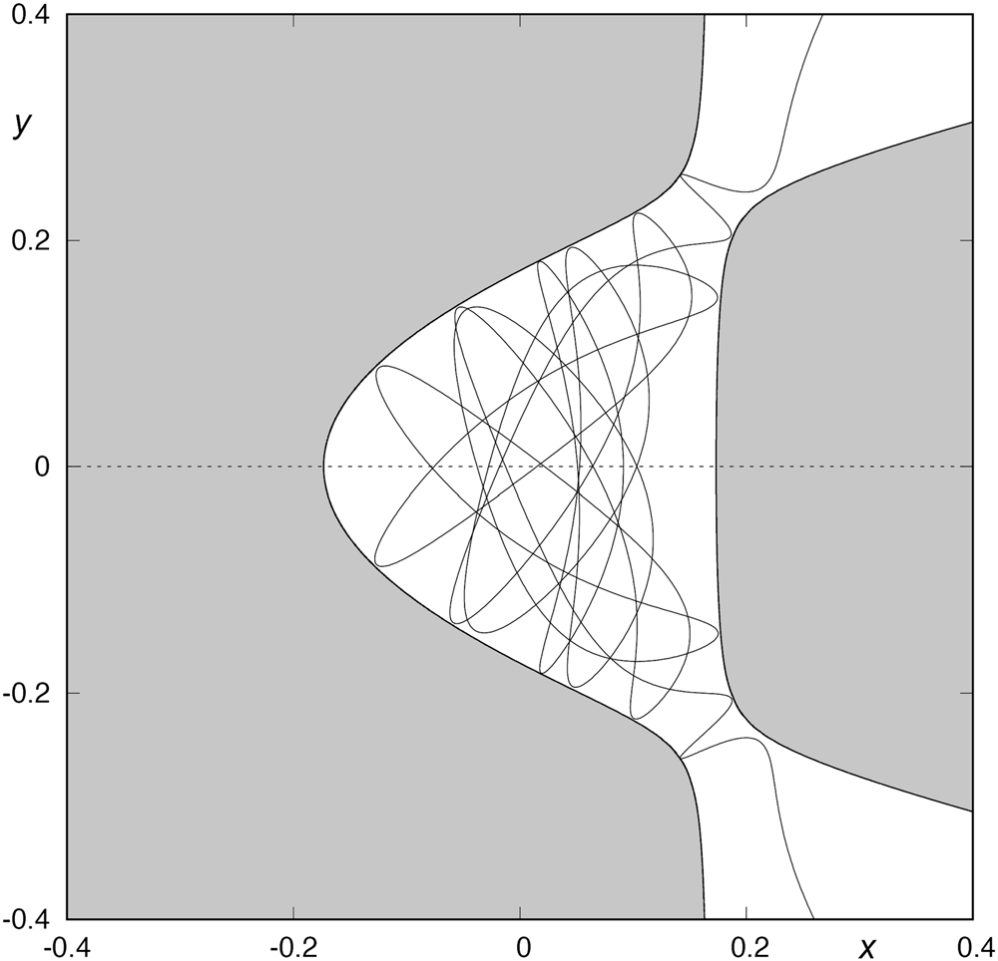
An orbit coming from the lower-right infinity and escaping through the upper-right window after fifteen intersections with the surface of section *y* = 0.

In Fig. 5, we show some of these sets, and we can observe that the sets *W*_*s*,3_(*ϕ*_*U*_), *W*_*s,*__7_(*ϕ*_*U*_) and *W*_*s*,11_(*ϕ*_*U*_) do not intersect *W*_*u*,1_(*ϕ*_*L*_). However, the intersection between *W*_*s*,15_(*ϕ*_*U*_) and *W*_*u*,1_(*ϕ*_*L*_) is not empty. This fact means that we can find orbits coming from the lower-right infinity and escaping through the upper-right window after fifteen intersections with the surface of section *y* = 0. We show an example of such an orbit in Fig. 6. We can also conclude that there are no orbits coming from the lower-right infinity and escaping through the upper-right window intersecting the axis *y* = 0 a number of times smaller than 15.

**Figure 7:**
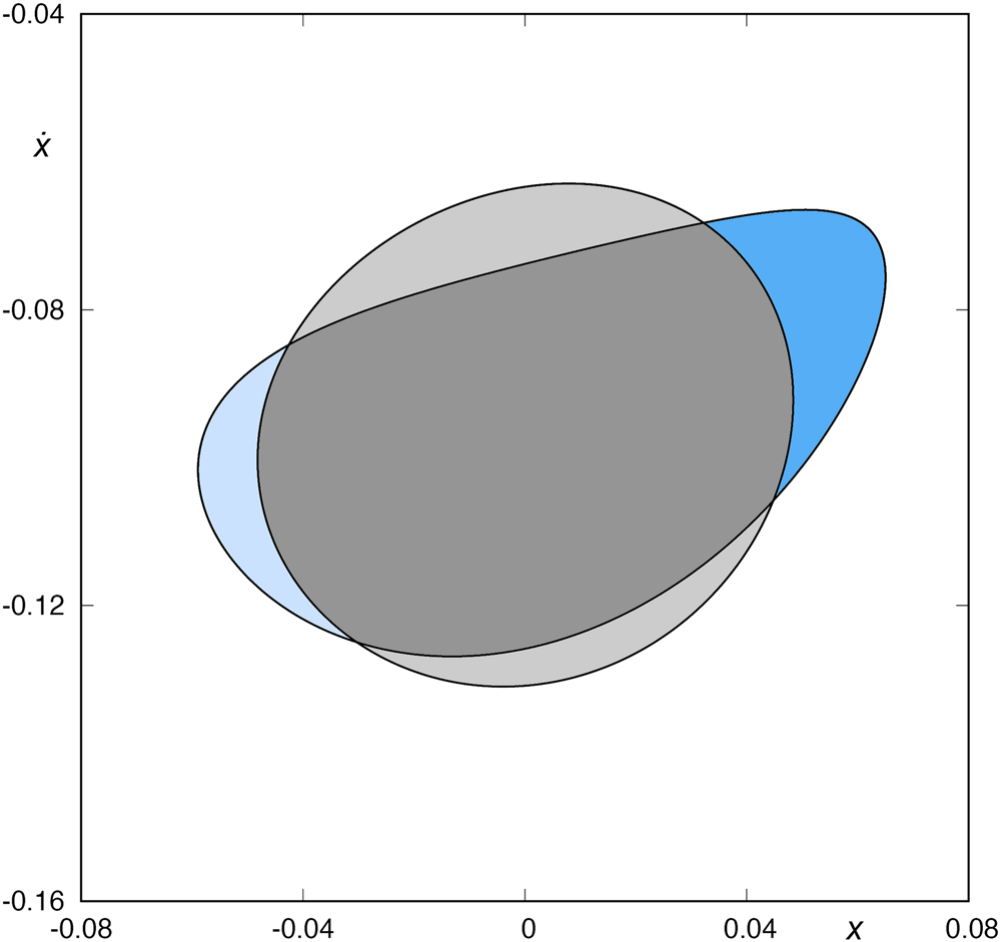
*W*_*s*,2_(*ϕ*_*U*_) and *W*_*u*,1_(*ϕ*_*U*_) for *h* = 0.01504. There are four intersection points related to homoclinic orbits to the upper-right guardian periodic orbit *ϕ*_*U*_(*t*).

**Figure 8:**
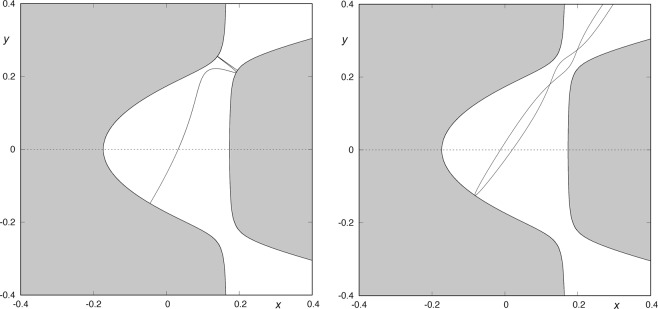
Homoclinic orbit (left panel) to *ϕ*_*U*_(*t*) intersecting the surface of section *y* = 0 two times, and orbit coming from the upper-right infinity, and going to the upper-right infinity, after two intersections with the surface of section *y* = 0 (right panel).

Now, we will focus our attention in the origin of the infinite spirals around *W*_*s*,3_(*ϕ*_*U*_) that we can observe in the upper-left panel of Fig. 5. To this purpose, we consider the intersections between the stable and unstable manifolds to the upper-right periodic orbit. In Fig. 7, we show the intersection between *W*_*s*,2_(*ϕ*_*U*_) and *W*_*u*,1_(*ϕ*_*U*_) in the surface of section *y* = 0 (with $\dot{y} < 0$), for *h* = 0.01504. We observe that *W*_*s*,2_(*ϕ*_*U*_) ∩ *W*_*u*,1_(*ϕ*_*U*_) ≠ $\varnothing$, that is, the two “rings” intersect. The intersection points correspond to homoclinic orbits to the upper-right periodic orbit *ϕ*_*U*_(*t*). These four homoclinic orbits intersect two times the surface of section *y* = 0. In Fig. 8 (left panel), we show one of these homoclinic orbits to *ϕ*_*U*_(*t*). Orbits starting inside both rings (colored in dark grey in Fig. 7) are coming from the upper-right infinity and are going to the upper-right infinity. They just pass through the center of the galaxy, intersecting the surface of section *y* = 0 two times. In Fig. 8 (right panel), we show one of these orbits. Moreover, orbits starting from one of the two crescents, colored in blue in Fig. 7, have a previous intersection with the surface of section *y* = 0, and of course escape in the future by the upper-right opening of the potential.

**Figure 9:**
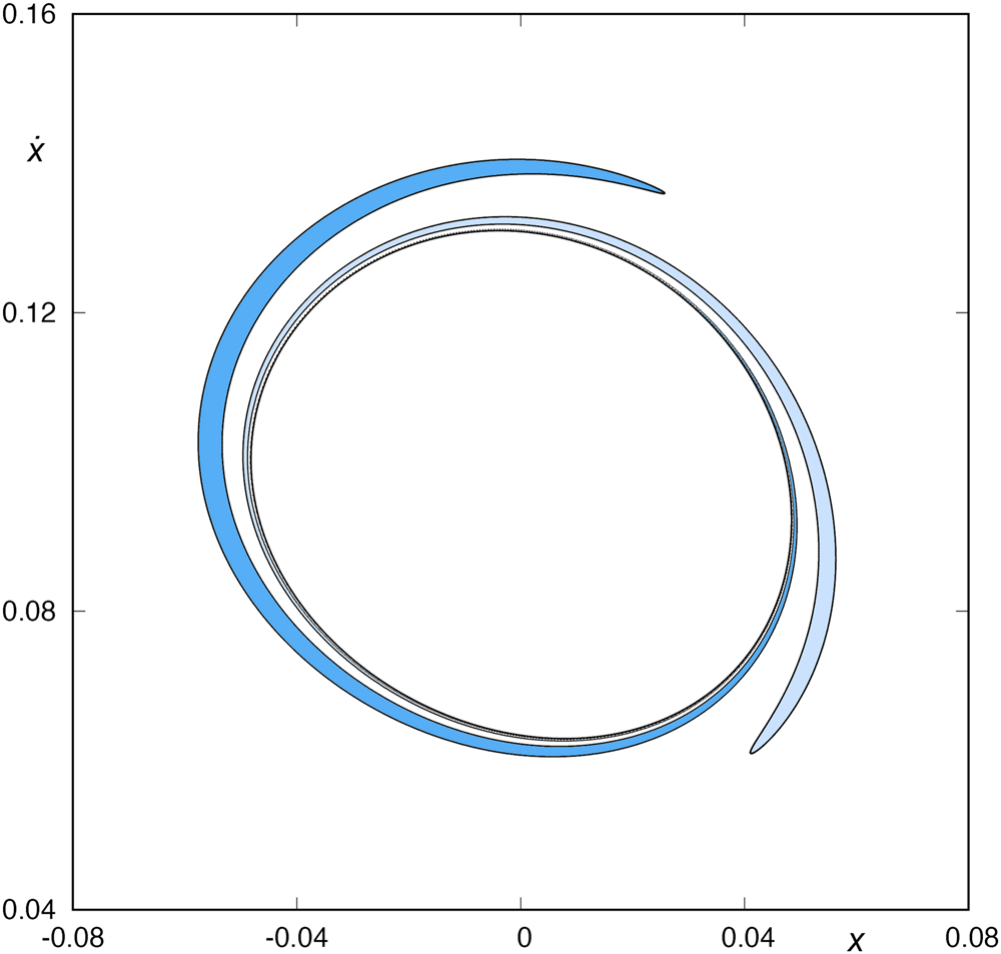
The third intersection *W*_*s*,3_(*ϕ*_*U*_) is composed of two tongues which spiral around the stable manifold to the first quadrant guardian orbit, *W*_*s*,1_(*ϕ*_*U*_). These two tongues are images of the two crescents colored in blue in Fig. 8.

**Figure 10:**
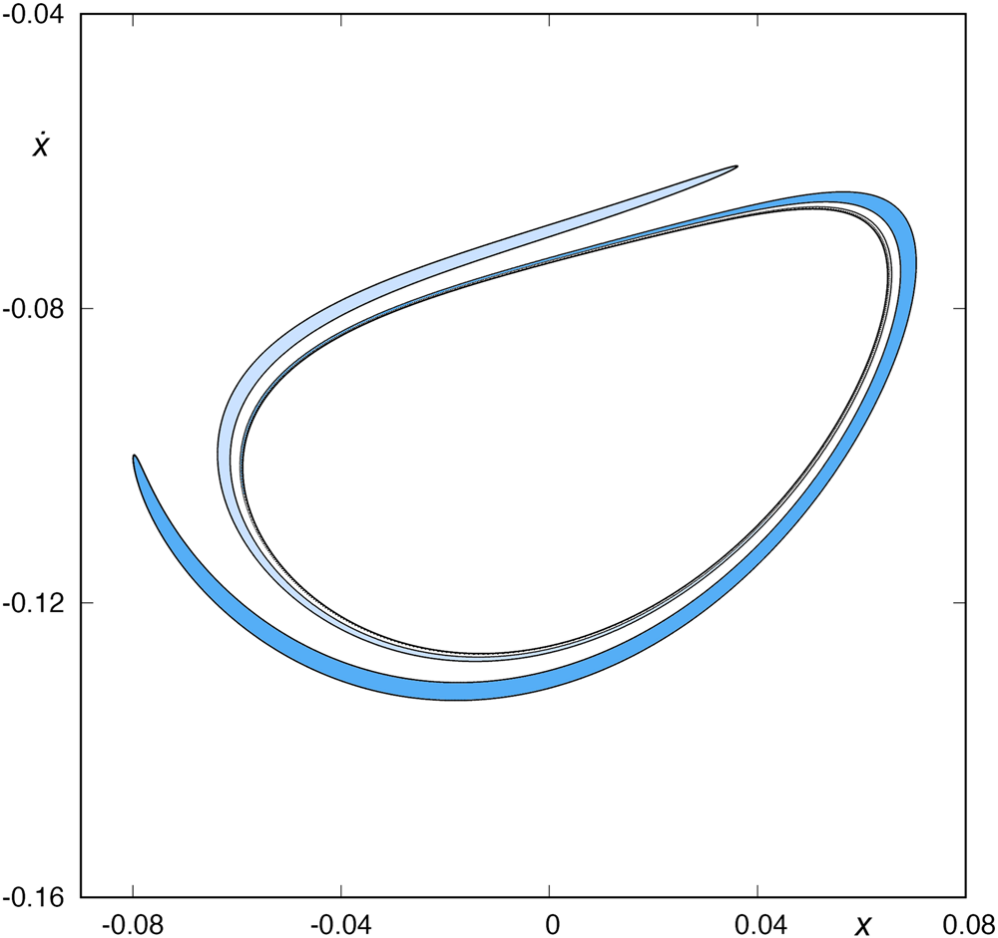
Trace of the fourth intersection of the stable manifold to *ϕ*_*U*_(*t*), *W*_*s*,4_(*ϕ*_*U*_), with the surface of section.

The third intersection, shown in Fig. 9, is composed of two “tongues” which spiral around the stable manifold to the first quadrant guardian orbit, *W*_*s*,1_(*ϕ*_*U*_). These two tongues are images of the two crescents colored in blue in Fig. 7. The spirals are infinite, but of course we have computed (and shown in Fig. 9) only a part of them. As we have mentioned above, *W*_*s*,3_(*ϕ*_*U*_(*t*)) does not intersect *W*_*u*,1_(*ϕ*_*U*_) or *W*_*u*,1_(*ϕ*_*L*_). Thus, all the orbits starting from these two tongues have a previous intersection with the surface of section *y* = 0, and will leave the galaxy by the upper-right window after two crossings with the axis *y* = 0. As a consequence of this fact, the fourth intersection of the stable manifold to the upper-right periodic orbit *ϕ*_*U*_(*t*) with the surface of section *y* = 0, is also composed of two tongues, which spiral around *W*_*s*,2_(*ϕ*_*U*_). In Fig. 10, we show the trace of the fourth intersection of the stable manifold to *ϕ*_*U*_(*t*) with the surface of section. As in the previous intersection, orbits starting inside these tongues will leave the galaxy by the upper-right window after three crossings with the axis *y* = 0. As before, *W*_*s*,4_(*ϕ*_*U*_) is composed of two “tongues” which spiral around the stable manifold to the first quadrant guardian orbit. These two tongues are images of the two crescents colored in blue in Fig. 9.

**Figure 11:**
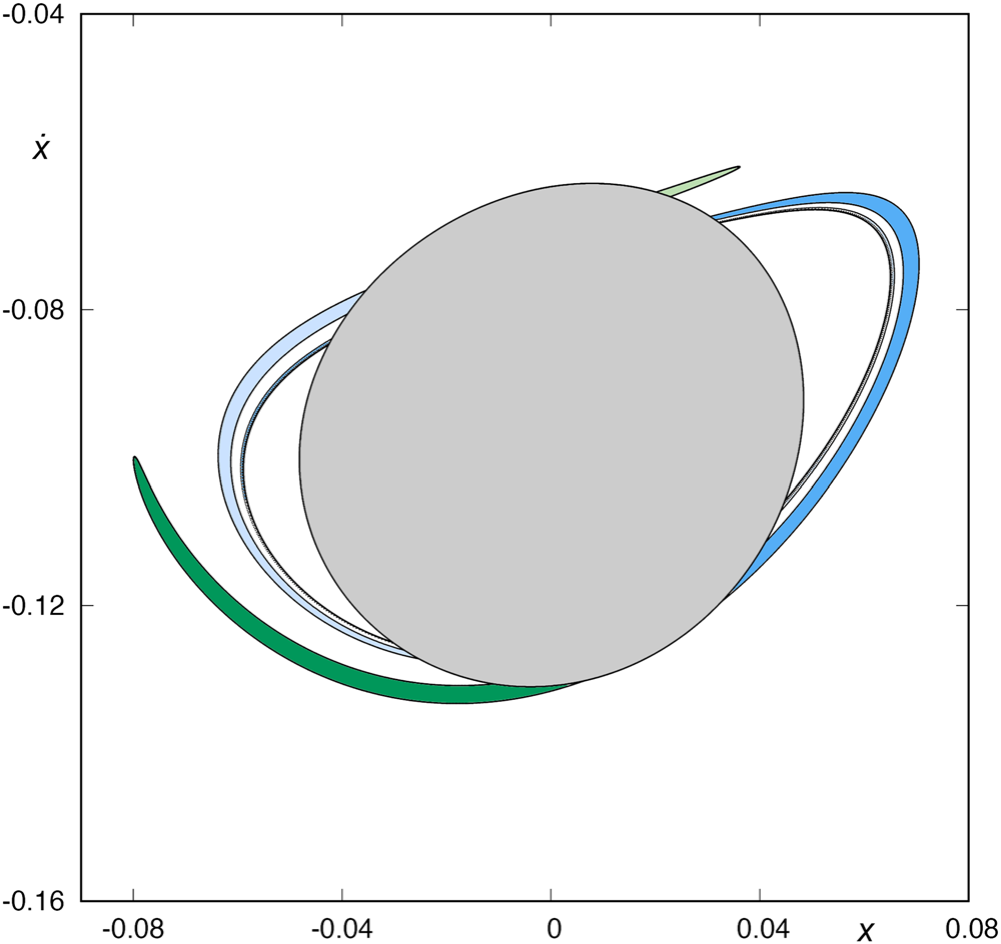
Intersection between *W*_*s*,4_(*ϕ*_*U*_) and *W*_*u*,1_(*ϕ*_*U*_), for *h* = 0.01504.

In Fig. 11, we show the intersection between *W*_*s*,4_(*ϕ*_*U*_) and *W*_*u*,1_(*ϕ*_*U*_). We observe that *W*_*s*,4_(*ϕ*_*U*_) intersects *W*_*u*,1_(*ϕ*_*U*_) at an infinite set of points. Each of these points of intersection between both manifolds corresponds to a homoclinic orbit to *ϕ*_*U*_(*t*) which intersects the surface of section *y* = 0 four times. Orbits starting inside *W*_1*,u*_(*ϕ*_*U*_), and inside one of the two tongues of *W*_*s*,4_(*ϕ*_*U*_), come from infinity by the upper-right window and leave the galaxy by the same window after four crossings of the axis *y* = 0. Orbits starting inside the tongues but outside the grey area enclosed by *W*_*u*,1_(*ϕ*_*U*_) have a previous intersection with the surface of section. We have colored these parts of the tongues in different colors in order to follow these orbits backward easily.

**Figure 12:**
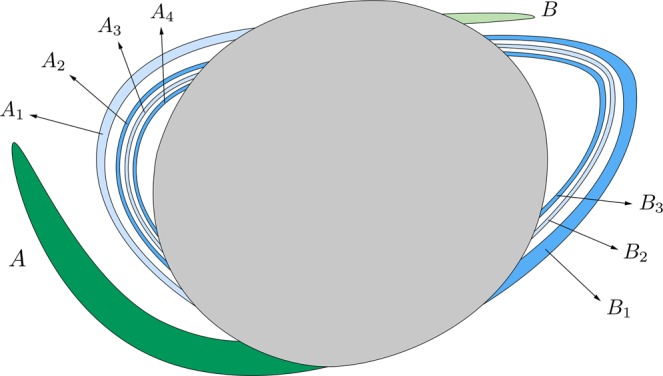
Scheme of the structure of *W*_*s*,4_(*ϕ*_*U*_) outside *W*_*u*,1_(*ϕ*_*U*_), for *h* = 0.01504.

In Fig. 12, we give an scheme of the structure of the part of *W*_*s*,4_(*ϕ*_*U*_) outside *W*_*u*,1_(*ϕ*_*U*_), in order to clarify the destiny of the different components of the tongues when we follow them backward in time. First, we will focus our attention in the left component of the manifolds, and the same analysis will be applied to the right component. We observe that we can distinguish two parts in the left component: the tongue named *A* and colored in dark green in Fig. 12, and an infinite set of “bridges”, each of them inside the previous one. We have numbered these bridges as shown in Fig. 12: *A*_1_, *A*_2_, *A*_3_, …. This set of bridges are alternately colored in light and medium blue, depending on the tongue of *W*_*s*,4_(*ϕ*_*U*_) they belong (see Fig. 10). The tongue *A* intersects *W*_*u*,1_(*ϕ*_*U*_) at two different points, corresponding to a pair of homoclinic orbits to *ϕ*_*U*_. These two orbits intersect the surface of section *y* = 0 four times. On the other hand, each bridge *A*_*ν*_, for any *ν* ∈ *N*, intersects *W*_*u*,1_(*ϕ*_*U*_) at four points, each of them corresponding to a homoclinic orbit to *ϕ*_*U*_, which intersects four times the surface of section *y* = 0. This means that there exists an infinite set of initial conditions corresponding to homoclinic orbits to *ϕ*_*U*_. Each of these set of homoclinic orbits intersects four times the surface of section *y* = 0.

The structure of the right component of the part of *W*_*s*,4_(*ϕ*_*U*_) outside *W*_*u*,1_(*ϕ*_*U*_) is similar. There is a tongue called *B* and colored in light green, and an infinite set of bridges (*B*_1_, *B*_2_, …) alternately colored in light and medium blue, as in the left component. The tongue *B* intersects *W*_*u*,1_(*ϕ*_*U*_) at two points, corresponding to a pair of homoclinic orbits to *ϕ*_*U*_. These two homoclinic orbits intersect the surface of section *y* = 0 four times. On the other hand, each bridge *B*_*ν*_, for any *ν* ∈ *N*, intersects *W*_*u*,1_(*ϕ*_*U*_) at four different points, each of them corresponding to a homoclinic orbit to *ϕ*_*U*_, which intersects four times the surface of section *y* = 0. Thus, there exists a second infinite set of initial conditions corresponding to homoclinic orbits to *ϕ*_*U*_, each one of them intersecting four times the surface of section *y* = 0.

Let us introduce here the notation *T* to denote the function that maps an initial condition in the surface of section *y* = 0 to the previous intersection of the corresponding orbit with the surface of section.

**Figure 13:**
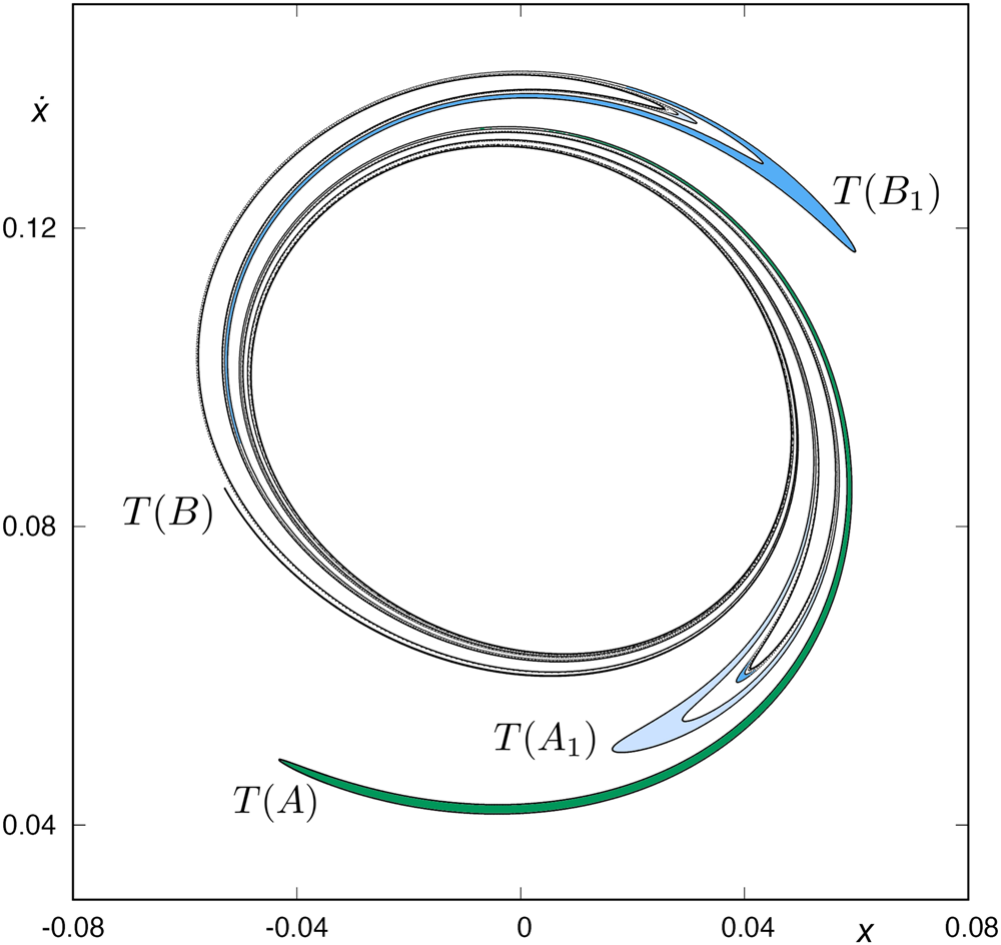
Trace of *W*_*s,*__5_(*ϕ*_*U*_), the fifth intersection of the stable manifold to *ϕ*_*U*_(*t*) with the surface of section *y* = 0, for *h* = 0.01504.

As commented above, the orbits with initial conditions inside *A*, *B*, *A*_*ν*_ and *B*_*ν*_, for any *ν* ∈ *N*, have a previous intersection with the surface of section *y* = 0. Following these orbits backward, we compute the fifth section (see Fig. 13). The intersection *W*_*s,*__5_(*ϕ*_*U*_) is composed by two simple tongues (colored in light and dark green) plus two complex ones. The simple ones are pre-images of the crescents *A* and *B*, shown in Fig. 12 and, thus, we denote them by *T*(*A*) and *T*(*B*), respectively. The complex tongues are composed of “subtongues” embedded inside each other, as “russian dolls”, following the same architecture than the bridges we have described in Fig. 12. There are an infinity of these subtongues infinitely winding around *W*_*s*,3_(*ϕ*_*U*_), the third intersection of the stable manifold to *ϕ*_*U*_ with the surface of section *y* = 0.

**Figure 14:**
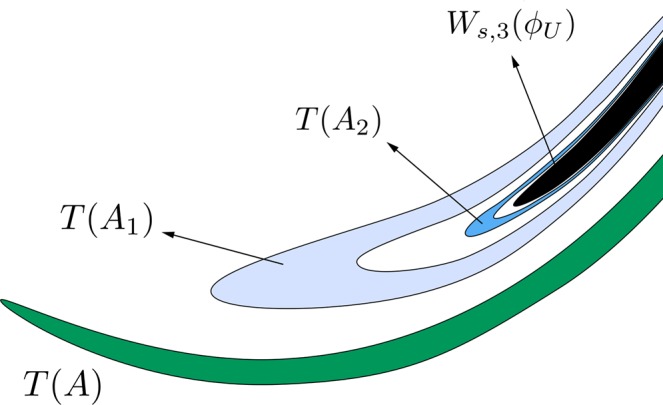
Scheme of a detail of the structure of *W*_*s,*__5_(*ϕ*_*U*_), for *h* = 0.01504.

In Fig. 14, we show a detail of the spiral structures we have found and shown in Fig. 13. Orbits starting inside the black region (corresponding to initial conditions inside *W*_*s*,3_(*ϕ*_*U*_)) escape after two intersections with the surface of section *y* = 0.

The orbits which start inside the complex tongues have a different fate depending on the parity of the number of subtongues in which they are embedded. Those starting from the colored area (Figs 13 and 14) are mapped on the pieces of the tongues analyzed in Fig. 12 which are not inside the grey area, and thus escape after four crossings of the surface of section. In Fig. 14, we have indicated the corresponding parts of the tongues in Fig. 12. There, *T*(*A*_1_) denotes the pre-image of the bridge *A*_1_, *T*(*A*_2_) the pre-image of the bridge *A*_2_, and so on.

**Figure 15:**
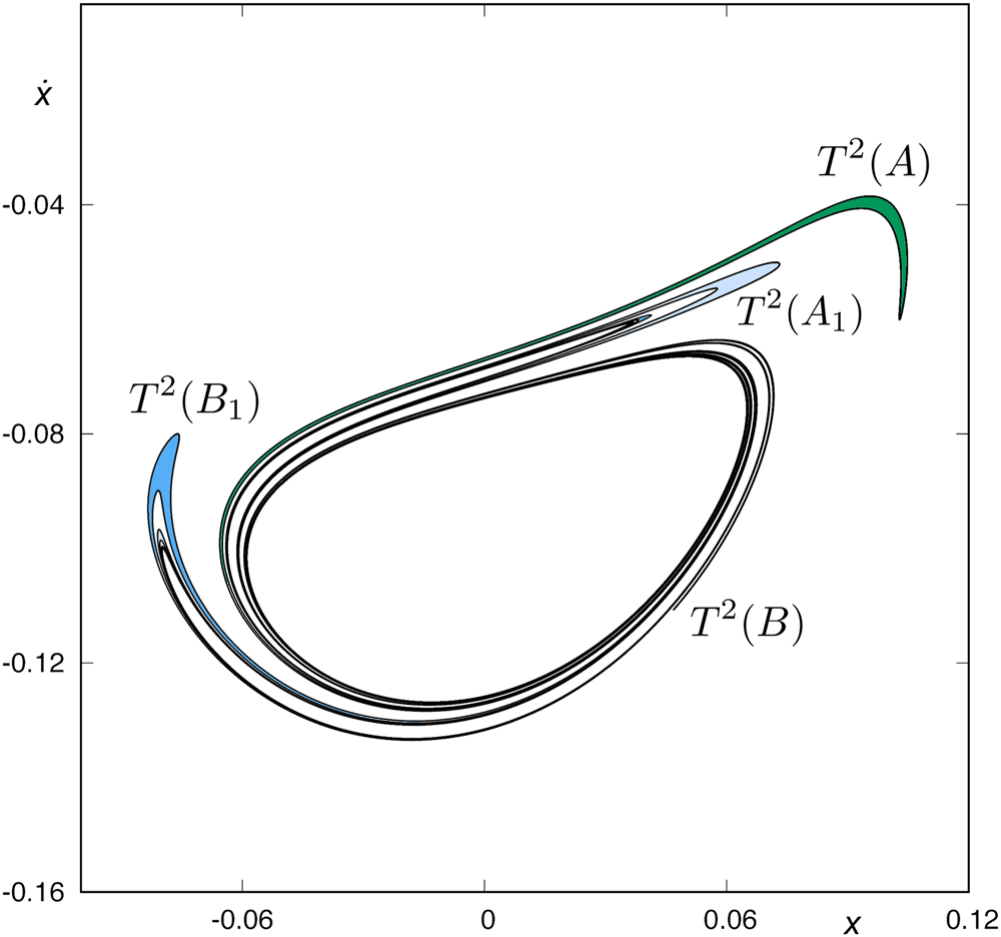
Trace of *W*_*s*,6_(*ϕ*_*U*_), the sixth intersection of the stable manifold to *ϕ*_*U*_(*t*) with the surface of section *y* = 0, for *h* = 0.01504.

**Figure 16:**
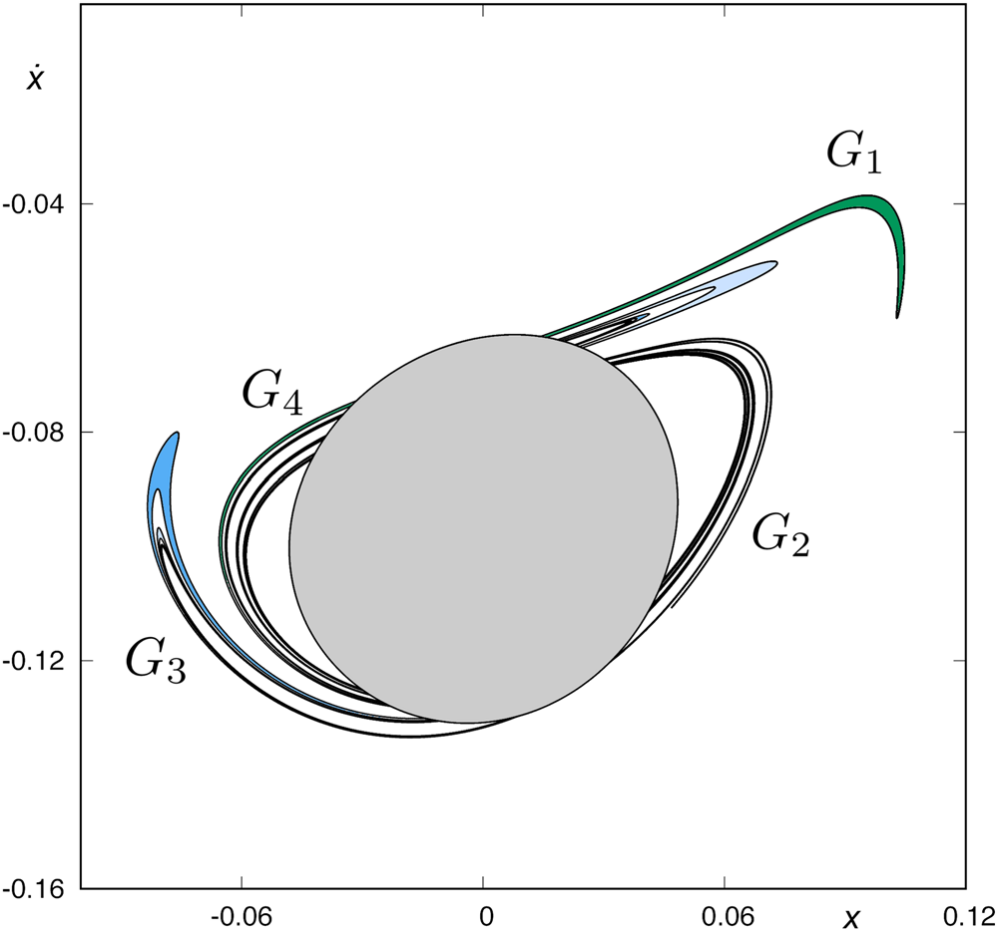
Intersection between *W*_*s*,6_(*ϕ*_*U*_) and *W*_*u*,1_(*ϕ*_*U*_), for *h* = 0.01504.

As *W*_*s,*__5_(*ϕ*_*U*_) (*y* = 0, $\dot{y} > 0$) does not intersect *W*_*u*,1_(*ϕ*_*U*_) (*y* = 0, $\dot{y} < 0$) or *W*_*u*,1_(*ϕ*_*L*_), the sixth intersection between the stable manifold to *ϕ*_*U*_ and the surface of section *y* = 0 (that is, *W*_*s*,6_(*ϕ*_*U*_)) has the same structure than *W*_*s,*__5_(*ϕ*_*U*_), as we can observe in Fig. 15. Here, we find *T*^2^(*A*), the pre-image of the tongue *T*(*A*), which belongs to *W*_*s,*__5_(*ϕ*_*U*_), *T*^2^(*B*), the pre-image of *T*(*B*), and also the sequences *T*^2^(*A*_1_), *T*^2^(*A*_2_), … and *T*^2^(*B*_1_), *T*^2^(*B*_2_), …. In Fig. 16, we show the intersection between *W*_*s*,6_(*ϕ*_*U*_) and *W*_*u*,1_(*ϕ*_*U*_). In this figure, we observe that some parts of *W*_*s*,6_(*ϕ*_*U*_) stay outside *W*_*u*,1_(*ϕ*_*U*_). As in the previous intersection of the stable manifold, the orbits which start inside the complex tongues have a different fate depending on the parity of the number of subtongues in which they are embedded. Those starting from the colored area (light and medium blue) are mapped on the pieces of the tongues (with equal color) analyzed in Figs 13 and 14, and thus escape after five crossings of the surface of section.

The structure of the intersection between *W*_*s*,6_(*ϕ*_*U*_) and *W*_*u*,1_(*ϕ*_*U*_) is quite complex, and it must be analyzed carefully. To that end, we have constructed a simplified scheme of what we can observe in Fig. 16. In order to simplify this analysis, we have divided it into four groups: *G*_1_, *G*_2_, *G*_3_ and *G*_4_.

**Figure 17:**
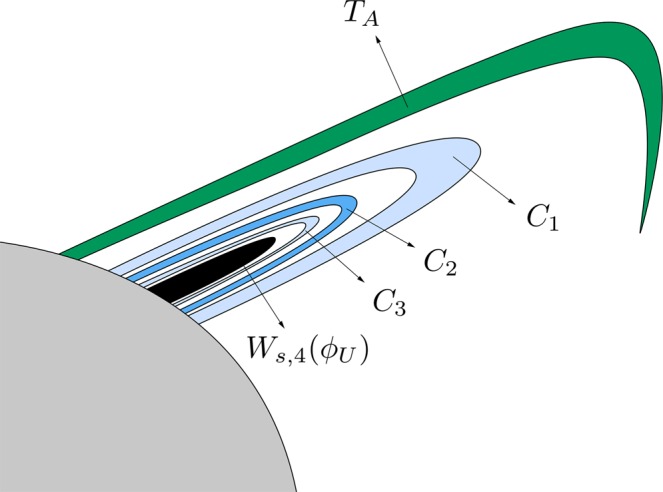
Detail of the structure of group *G*_1_ of the intersection between *W*_*s*,6_(*ϕ*_*U*_) and *W*_*u*,1_(*ϕ*_*U*_), for *h* = 0.01504.

*G*_1_ (Fig. 17) is composed of a simple tongue, colored in dark green and named *T*_*A*_, plus an infinite set of bridges which tends to *W*_*s*,4_(*ϕ*_*U*_) (colored in black in the figure). The tongue *T*_*A*_ is the part of *T*^2^(*A*) outside *W*_*u*,1_(*ϕ*_*U*_). The infinite set of bridges tending to *W*_*s*,4_(*ϕ*_*U*_) is formed by bridges of alternating colors (light and medium blue) depending on their origin in *W*_*s*,4_(*ϕ*_*U*_) (see Fig. 12). In Fig. 17, *C*_1_ represents the part of *T*^2^(*A*_1_) outside *W*_*u*,1_(*ϕ*_*U*_) and, in general, *C*_*ν*_ denotes the part of *T*^2^(*A*_*ν*_) outside *W*_*u*,1_(*ϕ*_*U*_), for any *ν* ∈ *N*. Each bridge of this infinite sequence intersects *W*_*u*,1_(*ϕ*_*U*_) at four points, which are the initial conditions of four homoclinic orbits to *ϕ*_*U*_. Each of these homoclinic orbits intersects six times the surface of section *y* = 0.

**Figure 18:**
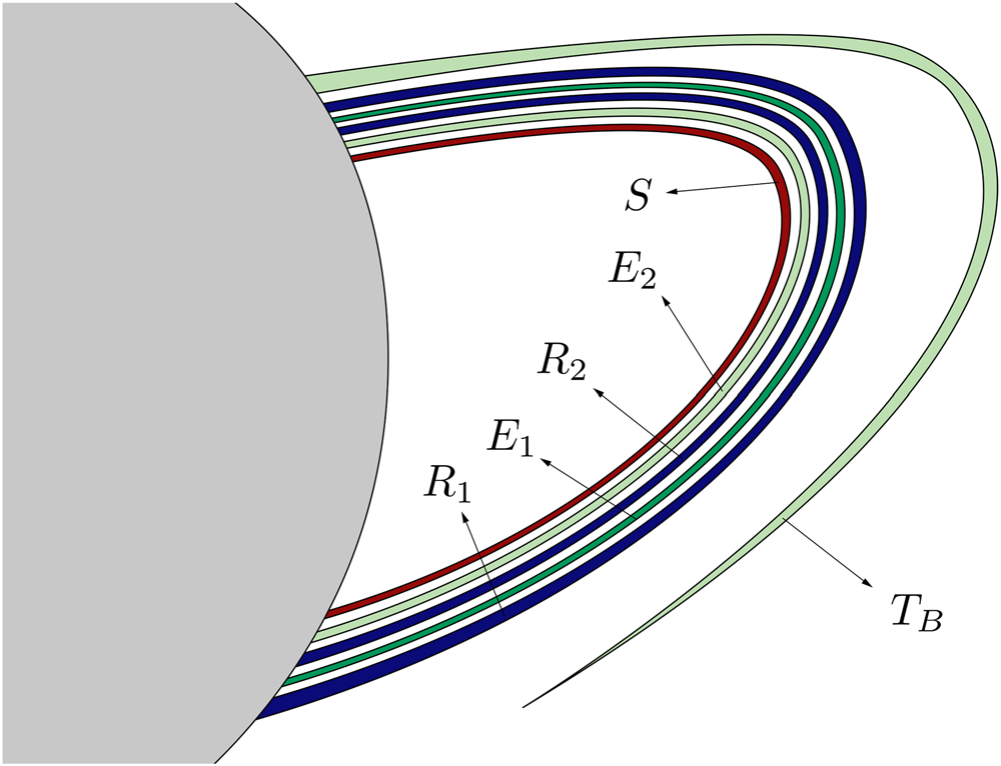
Detail of the structure of group *G*_2_ of the intersection between *W*_*s*,6_(*ϕ*_*U*_) and *W*_*u*,1_(*ϕ*_*U*_), for *h* = 0.01504.

**Figure 19:**
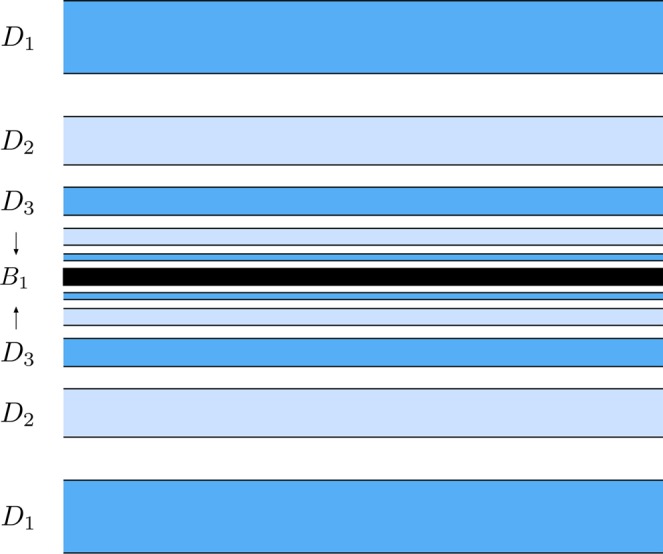
Detail of the composition of ring *R*_1_, belonging to the group *G*_2_ in the part of *W*_*s*,6_(*ϕ*_*U*_) outside *W*_*u*,1_(*ϕ*_*U*_).

*G*_2_ (Fig. 18) is composed of a simple tongue (colored in light green, and named *T*_*B*_), plus an infinite set of simple bridges and “rings”. The crescent *T*_*B*_ is part of *T*^2^(*B*). The infinite set of simple bridges and rings tends to *W*_*s*,4_(*ϕ*_*U*_), and it is composed of an infinite set of simple bridges (as those which appear in *W*_*s*,4_(*ϕ*_*U*_)) and a set of a new kind of structure called ring. We define a ring as an infinite set of bridges tending to a simple bridge belonging to a part of *W*_*s*,4_(*ϕ*_*U*_). In Fig. 18, we have represented a detail of this structure. The first ring, named *R*_1_, is a continuation of the group *G*_3_ (see Fig. 16). Figure 19 gives a detail of its composition. We observe that *R*_1_ is formed by two infinite sequences of bridges that accumulate at *B*_1_, which is one of the bridges belonging to the part of *W*_*s*,4_(*ϕ*_*U*_) outside *W*_*u*,1_(*ϕ*_*U*_). We have also indicated the origin of each of the bridges in *R*_1_, by writing the name of the bridges of *G*_3_ (*D*_1_, *D*_2_, *D*_3_, …) they come from. Each of the bridges belonging to one of these two sequences intersects *W*_*u*,1_(*ϕ*_*U*_) at four points. Each of these points is associated to the initial conditions of a homoclinic orbit to *ϕ*_*U*_, which intersects six times the surface of section *y* = 0.

Following with the analysis of *G*_2_, we find a bridge which corresponds to a part of *T*^2^(*A*). We have named this bridge *E*_1_ in Fig. 18. This bridge intersects *W*_*u*,1_(*ϕ*_*U*_) at four points, each of them corresponding to a homoclinic orbit to *ϕ*_*U*_ that intersetcs six times the surface of section *y* = 0.

**Figure 20:**
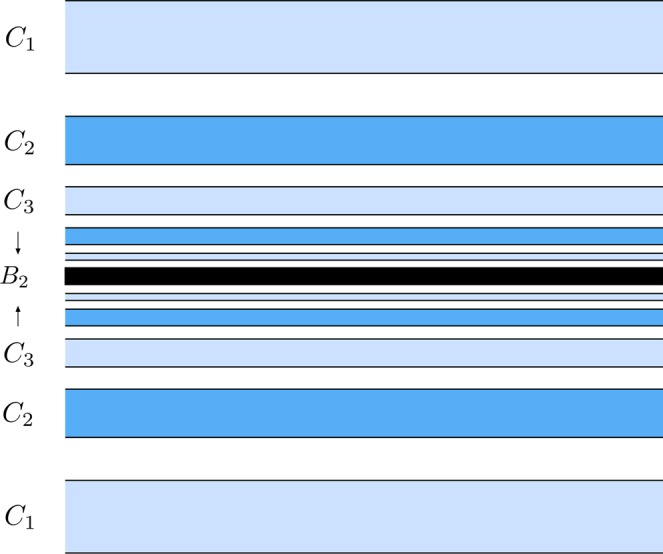
Detail of the composition of ring *R*_2_, belonging to the group *G*_2_ in the part of *W*_*s*,6_(*ϕ*_*U*_) outside *W*_*u*,1_(*ϕ*_*U*_).

Then, we find a second ring, named *R*_2_, inside the bridge *E*_1_. The composition of *R*_2_ is similar to that of *R*_1_. This second ring is a continuation of the infinite set of bridges which form *G*_1_. A detail of its structure is shown in Fig. 20. *R*_2_ is formed by two infinite sequences of bridges that accumulate at *B*_2_, which is one of the bridges belonging to the part of *W*_*s*,4_(*ϕ*_*U*_) outside *W*_*u*,1_(*ϕ*_*U*_). We have also indicated the origin of each of the bridges in *R*_2_, by writing the name of the bridges of *G*_1_ (*C*_1_, *C*_2_, *C*_3_, …) they come from. Each of the bridges belonging to one of these two sequences intersects *W*_*u*,1_(*ϕ*_*U*_) at four points. Each of these points is associated to the initial conditions of a homoclinic orbit to *ϕ*_*U*_, which intersects six times the surface of section *y* = 0.

Inside *R*_2_, there is a bridge which corresponds to a part of *T*^2^(*B*). We have named this bridge *E*_2_ in Fig. 18. This bridge intersects *W*_*u*,1_(*ϕ*_*U*_) at four points. Each of these points corresponds to a homoclinic orbit to *ϕ*_*U*_ that intersetcs six times the surface of section *y* = 0.

The union of these four structures (*R*_1_, *E*_1_, *R*_2_ and *E*_2_) is a new kind of object, named “belt”. Inside *E*_2_, we find a set colored in brown in Fig. 18, containing an infinite sequence of belts infinitely spiraling around *W*_*s*,4_(*ϕ*_*U*_). Each belt intersects *W*_*u*,1_(*ϕ*_*U*_) at an infinite set of points, each of them corresponding to a homoclinic orbit to *ϕ*_*U*_. As the homoclinics described before, all these orbits intersect six times the surface of section.

**Figure 21:**
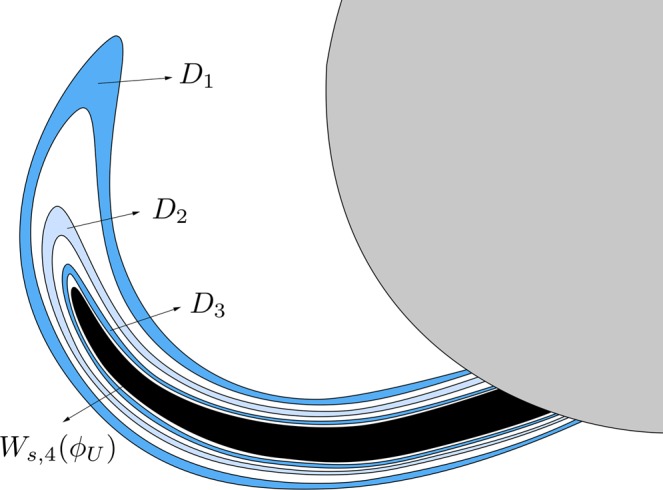
Detail of the structure of group *G*_3_ of the intersection between *W*_*s*,6_(*ϕ*_*U*_) and *W*_*u*,1_(*ϕ*_*U*_), for *h* = 0.01504.

The structure of *G*_3_ is shown in Fig. 21. *G*_3_ is composed of an infinite set of bridges which tends to *W*_*s*,4_(*ϕ*_*U*_) (colored in black in the figure). The infinite set of bridges tending to *W*_*s*,4_(*ϕ*_*U*_) is formed by bridges of alternating colors (medium and light blue) depending on their origin in *W*_*s*,4_(*ϕ*_*U*_) (see Fig. 12). In Fig. 21, *D*_1_ represents the part of *T*^2^(*B*_1_) outside *W*_*u*,1_(*ϕ*_*U*_) and, in general, *D*_*ν*_ denotes the part of *T*^2^(*B*_*ν*_) outside *W*_*u*,1_(*ϕ*_*U*_), for any *ν* ∈ *N*. Each bridge of this infinite sequence intersects *W*_*u*,1_(*ϕ*_*U*_) at four points, which are the initial conditions of a homoclinic orbit to *ϕ*_*U*_. Each of these homoclinic orbits intersects six times the surface of section *y* = 0.

**Figure 22:**
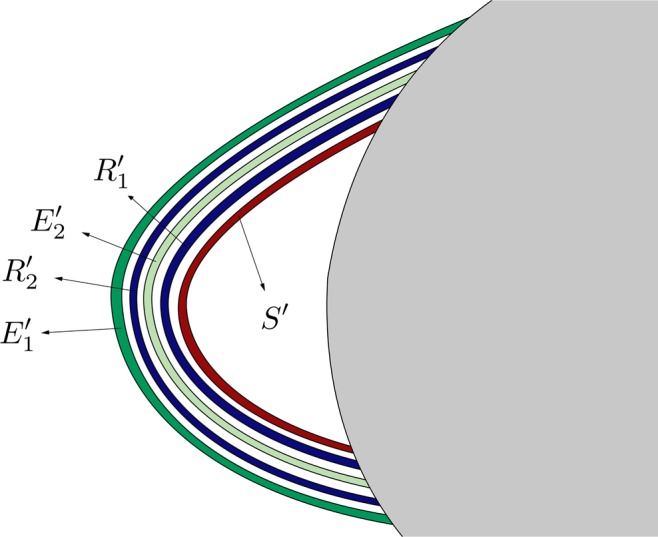
Detail of the structure of group *G*_4_ of the intersection between *W*_*s*,6_(*ϕ*_*U*_) and *W*_*u*,1_(*ϕ*_*U*_), for *h* = 0.01504.

*G*_4_ (Fig. 22) has a similar structure to that of *G*_2_. It is composed of an infinite set of belts. First, in the outer part of the structure, we find a bridge which corresponds to a part of *T*^2^(*A*). We have named this bridge *E*′_1_ in Fig. 22. This bridge intersects *W*_*u*,1_(*ϕ*_*U*_) at four points. Each of these points corresponds to a homoclinic orbit to *ϕ*_*U*_ that intersetcs six times the surface of section *y* = 0. Inside *E*′_1_, we find the ring *R*′_2_, with the same origin than *R*_2_, as *R*′_2_ is also a continuation of the infinite set of bridges that forms the group *G*_1_. Thus, it has the same composition than *R*_2_ (Fig. 20): two infinite sequences of bridges that accumulate at *B*_2_, which is one of the bridges belonging to the part of *W*_*s*,4_(*ϕ*_*U*_) outside *W*_*u*,1_(*ϕ*_*U*_). Each of the bridges belonging to one of these two sequences intersects *W*_*u*,1_(*ϕ*_*U*_) at four points, and each of these points is associated to the initial conditions of a homoclinic orbit to *ϕ*_*U*_, which intersects six times the surface of section *y* = 0.

Inside *R*′_2_, there is a bridge corresponding to a part of *T*^2^(*B*). We have named this bridge *E*′_2_ in Fig. 18. This bridge intersects *W*_*u*,1_(*ϕ*_*U*_) at four points. Each of these points corresponds to a homoclinic orbit to *ϕ*_*U*_ that intersetcs six times the surface of section *y* = 0.

Then, and inside *E*′_1_, there is a ring, named *R*′_1_, with the same structure than *R*_1_ (Fig. 19). *R*′_1_ is formed by two infinite sequences of bridges that accumulate at *B*_1_, which is one of the bridges belonging to the part of *W*_*s*,4_(*ϕ*_*U*_) outside *W*_*u*,1_(*ϕ*_*U*_). Each of the bridges belonging to one of these two sequences intersects *W*_*u*,1_(*ϕ*_*U*_) at four points, associated to the initial conditions of a homoclinic orbit to *ϕ*_*U*_, which intersects six times the surface of section *y* = 0.

As it occurs in *G*_2_, the union of these four structures (*E*′_1_, *R*′_2_, *E*′_2_ and *R*′_1_) repeats infinitely spiraling around *W*_*s*,4_(*ϕ*_4_). Each belt intersects *W*_*u*,1_(*ϕ*_*U*_) at an infinite set of points, each of them corresponding to a homoclinic orbit to *ϕ*_*U*_. As the homoclinics described before, all these orbits intersect six times the surface of section.

**Figure 23:**
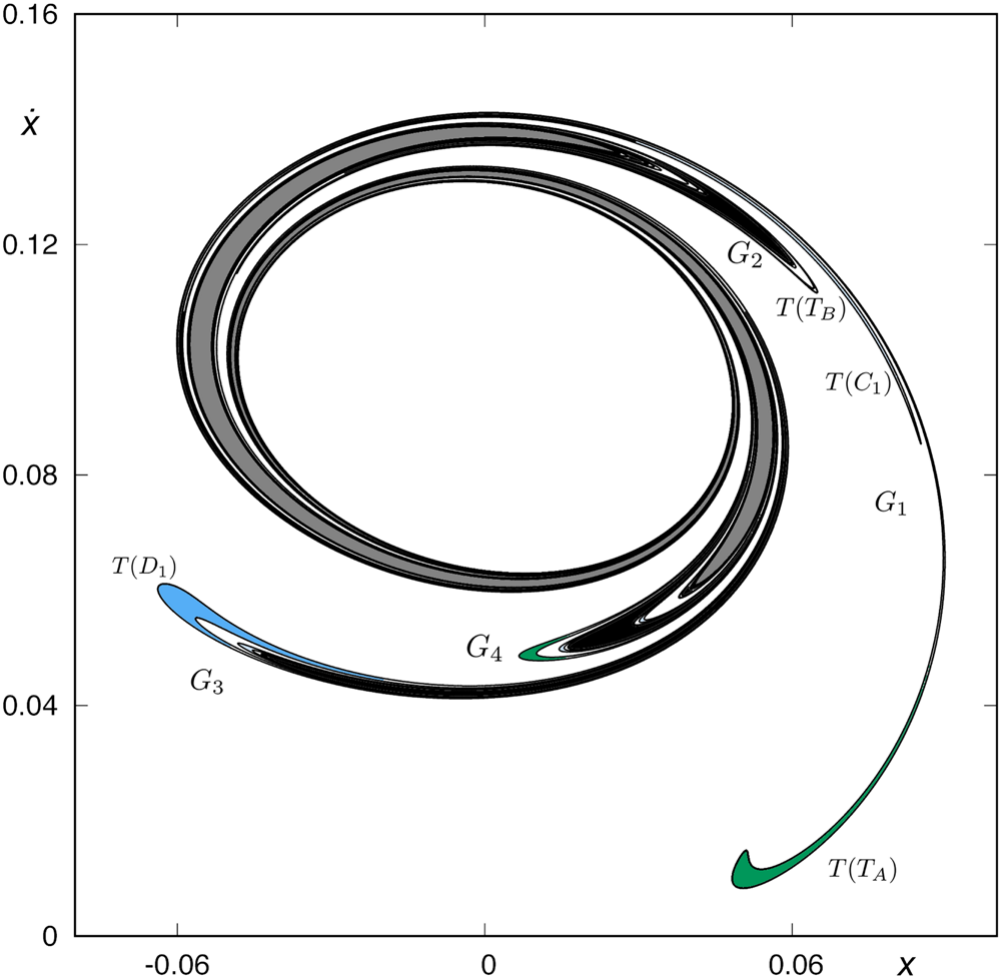
Trace of *W*_*s,*__7_(*ϕ*_*U*_), the seventh intersection of the stable manifold to *ϕ*_*U*_(*t*) with the surface of section *y* = 0, for *h* = 0.01504.

Orbits starting inside *W*_1*,u*_(*ϕ*_*U*_), and inside one of the colored areas of *W*_*s*,6_(*ϕ*_*U*_), come from infinity by the upper-right window and leave the galaxy by the same window after six crossings of the axis *y* = 0. Orbits starting inside the colored areas but outside the grey area enclosed by *W*_*u*,1_(*ϕ*_*U*_) have a previous intersection with the surface of section. Following these orbits backward, we compute the seventh section (see Fig. 23). We have colored in black the tongues belonging to *W*_*s,*__5_(*ϕ*_*U*_), and in dark grey the two tongues belonging to *W*_*s*,3_(*ϕ*_*U*_). Orbits with initial conditions inside the dark grey region (corresponding to *W*_*s*,3_(*ϕ*_*U*_)) escape after two intersections with the axis *y* = 0, and orbits starting inside the black region (corresponding to initial conditions inside *W*_*s,*__5_(*ϕ*_*U*_)) escape after four intersections with the surface of section *y* = 0. Finally, orbits starting in the colored regions escape after six intersections with the surface of section.

**Figure 24:**
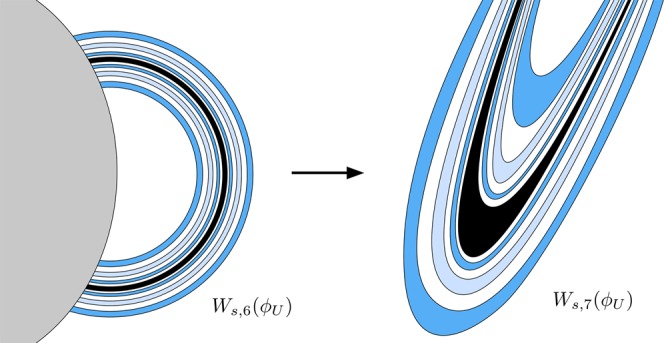
A ring structure in the part of *W*_*s*,6_(*ϕ*_*U*_) outside *W*_*u*,1_(*ϕ*_*U*_) (left) and a detail of its composition when we follow it backward until the seventh intersection with the surface of section (right). There are two infinite sequences of tongues infinitely accumulating at a tongue belonging to *W*_*s,*__5_(*ϕ*_*U*_), which is colored in black in the right sketch.

The structure of the intersection *W*_*s,*__7_(*ϕ*_*U*_) becomes more intrincately complex than that of *W*_*s*,6_(*ϕ*_*U*_). In general, we observe that for any tongue belonging to *W*_*s,*__5_(*ϕ*_*U*_), there is an infinite sequence of tongues in *W*_*s,*__7_(*ϕ*_*U*_) tending to it. This is due to the fact that each bridge belonging to *W*_*s*,6_(*ϕ*_*U*_) that intersects *W*_*u*,1_(*ϕ*_*U*_) originates a tongue infinitely spiraling around *W*_*s,*__5_(*ϕ*_*U*_) in *W*_*s,*__7_(*ϕ*_*U*_). Thus, each ring in *W*_*s*,6_(*ϕ*_*U*_) generates an infinite sequence of tongues tending to the corresponding tongue in *W*_*s,*__5_(*ϕ*_*U*_) when we obtain the seventh intersection of the stable manifold to *ϕ*_*U*_, in the same manner than we find an infinite sequence of bridges tending to a simple bridge in the ring structure. We have illustrated this mechanism in Fig. 24.

## Conclusions

In this paper, we study the properties of the escape of orbits from a potential presenting two channels of escape through the analysis of the asymptotic surfaces to the Lyapunov periodic orbits in the openings of the potential well. Depending on the value of the energy, the system exhibit different types of behavior. There is a critical value of the energy (*h*_*c*_) such that, for larger values of *h*, the curves of zero velocity are open and test particles may escape. For *h* < *h*_*c*_, the curves are effectively closed and particles originated from the central potential region are confined there. For each value of *h* larger than *h*_*c*_, there is an unstable periodic orbit located at every opening of the potential well. The asymptotic curves of the various unstable periodic orbits intersect at a complicated set of homoclinic and heteroclinic points, and govern the escape to infinity from the potential well. In our investigation, we have analyzed the geometry of these manifolds through the study of their successive intersections with the surface of section *y* = 0, in order to show the shape and the size of the windows through which test particles escape from the potential well.
